# Phrenic Nerve Conduction Reference Values in Healthy Adults: An Exploratory Cross-Sectional Study in a Mexican Population

**DOI:** 10.3390/clinpract15110209

**Published:** 2025-11-16

**Authors:** Francisco Javier González-López, Josefina Hernández-Cervantes, Sol Ramírez-Ochoa, Gabino Cervantes-Guevara, Guillermo A. Cervantes-Cardona, Francisco Javier Hernández-Mora, Berenice Vicente-Hernández, Alejandro González-Ojeda, Clotilde Fuentes-Orozco, Janet Cristina Vázquez-Beltrán, Enrique Cervantes-Pérez

**Affiliations:** 1 Department of Clinical Neurophysiology, Hospital Civil de Guadalajara Fray Antonio Alcalde, Guadalajara 44280, Jalisco, Mexico; pac_sagt_89@hotmail.com; 2 Department of Electrodiagnosis, Centro Médico Nacional 20 de Noviembre, ISSSTE, Mexico City 03104, Mexico; drajosef.hdz.cervantes@gmail.com; 3 Department of Internal Medicine, Hospital Civil de Guadalajara Fray Antonio Alcalde, University of Guadalajara, Guadalajara 44280, Jalisco, Mexico; ramirez_ochoa_sol@hotmail.com (S.R.-O.); dra.berenicevicente@gmail.com (B.V.-H.); 4 Department of Gastroenterology, Hospital Civil de Guadalajara Fray Antonio Alcalde, Guadalajara 44280, Jalisco, Mexico; gabino_guevara@hotmail.com; 5 Department of Welfare and Sustainable Development, Centro Universitario del Norte, University of Guadalajara, Colotlán 46200, Jalisco, Mexico; 6 Department of Philosophical, Methodological, and Instrumental Disciplines, Centro Universitario de Ciencias de la Salud, University of Guadalajara, Guadalajara 44340, Jalisco, Mexico; gacervantes66@gmail.com; 7 Department of Human Reproduction, Growth and Child Development, Health Sciences University Center, University of Guadalajara, Guadalajara 44280, Jalisco, Mexico; frank.gine@gmail.com; 8 Department of Obstetrics, Hospital Civil de Guadalajara Fray Antonio Alcalde, Guadalajara 44280, Jalisco, Mexico; 9 Biomedical Research Unit 02, Hospital de Especialidades, Centro Médico Nacional de Occidente, IMSS, Guadalajara 44350, Jalisco, Mexico; avygail5@gmail.com (A.G.-O.); clotilde.fuentes@gmail.com (C.F.-O.); 10 School of Medicine, Instituto Politécnico Nacional, Mexico City 11340, Mexico; janet.cris.beltran@gmail.com; 11 School of Medicine, Centro Universitario de Tlajomulco, University of Guadalajara, Tlajomulco de Zúñiga 45641, Jalisco, Mexico

**Keywords:** phrenic nerve conduction, normative data, electrophysiology, respiratory function, neuromuscular disorders

## Abstract

**Background/Objectives**: Phrenic nerve conduction (PNC) studies are essential for evaluating respiratory dysfunction and neuromuscular disorders. Despite international reference data, no normative values exist for the Mexican population. This study aimed to establish reference values for PNC latency and amplitude in healthy Mexican adults. **Methods**: We conducted a cross-sectional study between June 2022 and February 2023 including healthy adults (>18 years). Bilateral PNC studies were performed using surface electrodes and a 4-channel stimulation device. Latency and amplitude were recorded, and demographic and anthropometric data were collected. **Results**: Fifty subjects (22 women, 44%; 28 men, 56%) were enrolled. Mean latency was 6.10 ms (SD ± 1.48), and mean amplitude was 0.60 mV (SD ± 0.20). Significant differences were observed in left phrenic nerve latency between women and men (median 5.83 vs. 6.37 ms, *p* = 0.0348) and in amplitude between left and right phrenic nerves (0.55 vs. 0.65 mV, *p* = 0.0036). No significant correlations were found between latency and age or between amplitude and thoracic perimeter; however, the correlation coefficient suggests a positive relationship for both that should be confirmed in future studies with a larger sample size. **Conclusions**: This is the first report of PNC normative values in Mexican adults. Findings are consistent with international data and provide locally relevant reference values. Larger multicenter studies are warranted to validate and expand these results.

## 1. Introduction

Neuroconduction (NC) studies are noninvasive medical procedures that aid in the assessment of neuromuscular diseases by providing measurements of the neuromuscular junction, peripheral nerves, dorsal root ganglion, and anterior horn neurons [1].

Phrenic nerve conduction (PNC) studies have been used for several years to evaluate patients with respiratory failure and those with suspected neuromuscular pathologies [2,3]. PNC has been demonstrated to be a useful diagnostic tool for evaluating diaphragmatic dysfunction in diseases such as amyotrophic lateral sclerosis (ALS). Its potential as an adjuvant in predicting the need for mechanical ventilation in patients with neuromuscular diseases, including Guillain–Barré syndrome, has also been explored [4,5]. Since the motor innervation of the diaphragm is provided solely by the phrenic nerve, direct stimulation of the diaphragm is an easy-to-perform neurophysiological procedure that allows for the specific analysis of the diaphragm. By excluding other respiratory muscles, direct stimulation can discern the effect of the central nervous system (CNS) [6,7] without coactivating the contralateral phrenic nerve and its corresponding hemidiaphragm [8].

In recent years, multiple studies have established normative PNC values in healthy populations [9,10,11,12,13], providing reference parameters to identify abnormalities in conditions such as pulmonary disorders, neuromuscular diseases, and postoperative respiratory dysfunction [8]. PNC is performed by measuring the latency of the contractile motor response after proximal electrical stimulation in real time. This value is correlated with age, as well as the area and amplitude, which are both related to the thoracic perimeter. This can be accomplished using surface electrodes (a noninvasive and easy-to-place method) or needle electrodes, which are invasive and complex to place [8,9,10].

The study by Bushbacher et al. [11] continues to be one of the most commonly used studies to establish reference values for normal PNC parameters in general practice, even though various patient factors have been identified that can affect the results, such as age, gender, geographic location (i.e., altitude above sea level), ambient temperature, and patient height [1,13]. This study was conducted in a population of 25 North American patients, which limits broad extrapolation of its results to other populations and highlights the need for larger and more local studies to obtain phrenic nerve conduction reference values for specific populations.

Among the most recent larger studies, a 2019 study conducted in France by Vincent et al. analyzed PNC in 155 healthy subjects, with the aim of determining reference values for their population. The results obtained in the study were as follows: amplitude [0.38 Mv (millivolts), standard deviation (SD) ± 0.15], latency (6.59 ms (milliseconds), SD ± 0.82), area (3.05 ms/mV, SD ± 1.51) and duration (16.14 ms, SD ± 5.80). No significant differences in latency or amplitude were detected between the right and left nerves. On the other hand, they positively correlated latency with age and reported that latency, amplitude and area were significantly greater in men [13]. However, even studies such as that of Vincent et al. have been questioned regarding their extrapolation to other populations [14].

Recent research, including that of Ly DHM et al. [15], has identified novel variables that could influence neuroconduction values. These include metabolic variables such as urea and glycosylated hemoglobin, which are associated with glycemic control. In populations with a high prevalence of metabolic syndrome, such as the Mexican population, these variables, in addition to others related to weight and body fat distribution, assume particular significance. This is due to the fact that metabolic syndrome is frequently undiagnosed in these populations. Consequently, it is imperative to emphasize the importance of research that can provide reference values in neuroconduction studies for specific local populations [16].

To our knowledge, no studies have been conducted in Mexico to obtain normal values for PNC velocity in healthy Mexican subjects. Therefore, we believe that it is important to conduct studies that can provide values tailored to our patient population. The present study aimed to obtain phrenic nerve latency and amplitude values in adult Mexican patients from central Mexico and to describe the clinical and demographic characteristics of this population.

## 2. Materials and Methods

For this study, healthy male and female patients between 18 and 65 years of age were recruited between June 2022 and February 2023. They had to meet the following inclusion criterion: body mass index (BMI) less than 28 kg/m^2^—the decision was made to utilize this BMI value as a cut-off point, given the well-documented correlation between this parameter and a decrease in amplitude ranging from 20% to 40% among patients with obesity. This is particularly salient in the context of high fat percentage in body composition [17], Consequently, a conservative cut-off point between overweight (BMI > 25 kg/m^2^) and obesity (BMI > 30 kg/m^2^) was arbitrarily determined for the study, given the unavailability of precise body composition measurements. Other inclusion criteria were a clinically healthy determination based on the clinical evaluation performed by the physician serving as principal investigator, which implied the absence of any acute or chronic illness, and written informed consent prior to any study procedure. The exclusion criteria were patients with major thoracic deformities, pregnant or breastfeeding patients, patients with a history of cancer and/or chemotherapy, and patients for whom supine positioning for NC studies was contraindicated.

Patients were recruited through local advertising at the “Centro Medico Nacional 20 de Noviembre”. Once a volunteer contacted us expressing interest in participating in the study, they were scheduled for an initial visit to explain the study procedures. If the subject wished to participate, they signed a written informed consent prior to any study procedures. After signing the informed consent form, a medical history, vital signs, and anthropometric measurements were taken.

For PNC assessment, subjects were placed supine with the neck in a neutral or slightly extended position. Surface electrodes were positioned according to standard techniques described by Buschbacher [11] and Vincent et al. [13]. In brief, the G1 active electrode is placed two fingers above the xiphoid process on the midline of the thorax. The G2 reference electrode is placed on the anterior costal margin 16 cm away from the G1 electrode. This setup allows for recording of diaphragmatic activity. The diaphragm is a muscle innervated by the phrenic nerve. The stimulus is applied to the posterior aspect of the sternocleidomastoid (SCM) muscle. The stimulation point is approximately three centimeters above the clavicle, where the phrenic nerve begins its descent into the thorax. A ground electrode was placed on the sternum. Neuroconduction was performed using a 4-channel Nicolet Viking Quest device (Natus Medical, Middleton, WI, USA) with reusable gold-plated surface electrodes, the equipment was calibrated according to the manufacturer’s recommendations, and the results were recorded in a logbook. The electrode arrays included stimulating, recording, and ground electrodes, as well as a temperature sensor. The device autonomously assessed skin impedance and determined the minimum stimulator current necessary for a supramaximal stimulus with an amplitude ranging from 10 to 100 mA and a duration ranging from 100 to 500 µs. This stimulus produced a “hiccup” sensation at the end of the breathing cycle. The filter operated within the 20 Hz to 10 kHz frequency range. Following a minimum of two supramaximal stimulations, the response with the highest baseline-to-peak amplitude was selected for each side. Latency was assessed from the beginning of the negative peak. Co-stimulation of the brachial plexus was observed during arm movement and mitigated with a minor adjustment to the stimulating electrode. The measuring device automatically analyzes the waveforms to correct for temperature and eliminate stimulus artifacts. The waveforms should have the following characteristics: clear, identifiable polarity; consistent configuration; defined onset; distinctive peaks; and a clear return to baseline. All measurements were performed by the same operator (FJGL). The medical office where the measurements were taken has air conditioning that is maintained at a fixed 24 °C and no other means of ventilation. However, the ambient temperature was not strictly measured.

The data obtained were stored in a Microsoft Excel 2021 database (Microsoft Corporation, Washington, DC, USA) for subsequent statistical analysis.

For statistical analysis, GraphPad Prism (version 9.3.1.471, GraphPad by Dotmatics, Woburn, MA, USA) was used. Descriptive statistics of the nominal qualitative variables, both absolute and relative frequencies, were calculated, and the data are presented as percentages and proportions. For quantitative variables such as age, height, weight, BMI, chest circumference, vital signs, amplitude, and latency, measures of central tendency and dispersion (mean, median, and standard deviation) were used.

With respect to the inferential statistics of the quantitative variables, their normality in the Gaussian distribution was first assessed using the D’Angostino—Pearson and Kolmogorov—Smirnov tests. Owing to the nonnormal distribution of the data, comparisons between groups were performed using the nonparametric Mann-Whitney U test. Inferential analysis was performed to compare latency and amplitude for the right and left phrenic nerves [18] and to compare measurements between sexes.

Finally, to obtain correlations between age and latency and between chest circumference and amplitude, simple linear regression was performed for each analysis.

In all analyses, *p* values less than 0.05 with a 95% confidence interval (CI) were considered significant.

This study was conducted in accordance with the Declaration of Helsinki and Good Clinical Practice (GCP) guidelines. The study protocol was reviewed and approved by the research and research ethics committees of the Centro Médico Nacional 20 de Noviembre (approval code: 086.2023). Written informed consent was obtained from all participants included in the study prior to any procedure being performed.

## 3. Results

A total of 50 healthy Mexican subjects were recruited, and their left and right PNC measurements were taken. Twenty-two women (44%) and 28 men (56%) were included. Table 1 shows the general characteristics of the subjects included in the study.

**Figure 1:**
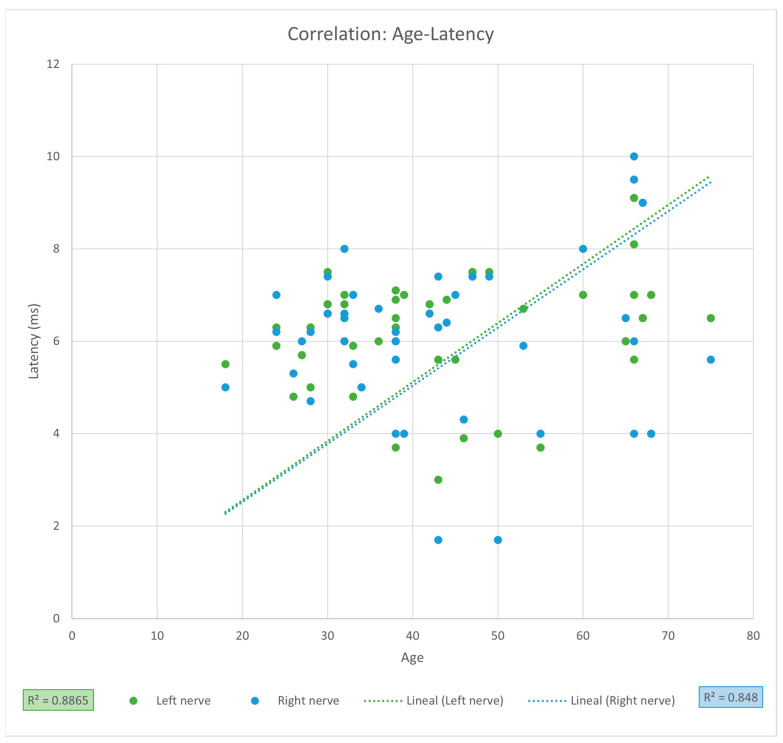
Linear regression of age and latency of phrenic nerve conduction. R^2^: coefficient of determination calculated from Person’s r.

**Figure 2:**
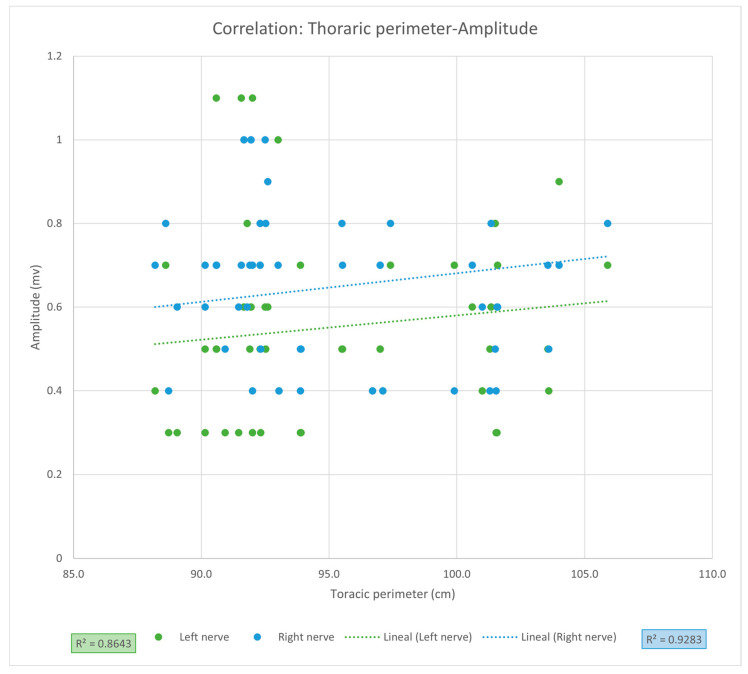
Linear regression of the thoracic perimeter-amplitude of phrenic nerve conduction. R^2^: coefficient of determination calculated from Pearson’s r.

For the PNC measurements, the mean latency was 6.10 ms (SD ± 1.48), and the mean amplitude was 0.60 mV (SD ± 0.20) for the entire sample. Comparison between left and right phrenic nerves showed a significantly greater amplitude on the right side (0.65 vs. 0.55 mV, *p* = 0.0036), while latencies were similar (Table 2). When comparing sexes, men exhibited significantly higher latency values for the left phrenic nerve (6.37 vs. 5.83 ms, *p* = 0.0348) (Table 3). Linear regression analysis revealed no significant association between PNC latency and age (LPN *r* = 0.9415, CI [0.8987, 1.2334], *r*^2^ = 0.8865, *p* = 0.1695; RPN *r* = 0.9208, CI [0.8639, 1.3671], r^2^ = 0.848, *p* = 0.5206) (Figure 1), nor between PNC amplitude and thoracic circumference (LPN r = 0.9297, CI [0.8787, 1.4153], *r*^2^ = 0.8643, *p* = 0.7169; RPN *r* = 0.9635, CI [0.9362, 1.1968], *r*^2^ = 0.9283, *p* = 0.2101, Figure 2). Despite high correlation coefficients, these results should be interpreted as strong trends without statistical significance, requiring validation in larger cohorts.

## 4. Discussion

Although the diagnostic and therapeutic importance of phrenic nerve latency and amplitude measurements is recognized, there are no standardized measures of normality for specific populations. The present findings are relevant as they provide the first normative PNC data reported in Mexican adults, offering a local reference framework that complements previously published international studies.

Our results show that the median latency (LPN 6.13 ms; RPN 6.07 ms) and amplitude (LPN 0.55 mV; RPN 0.65 mV) of both phrenic nerves are within the normal range, as reported by Buschbacher [11]. Davis et al. [12] evaluated 18 healthy British subjects and reported a mean latency of 7.7 ms and amplitude range of 0.16–0.50 mV. In contrast to the population in this study, some of their volunteers had significantly reduced amplitudes, although the latency was similar. In a British patient population, Mier et al. reported only means for the latency of both phrenic nerves of 84 healthy individuals: 6.94 ms for the right and 6.61 ms for the left [19]. In Portugal, Carvalho et al. included 18 disease-free individuals with a mean latency of 7.7 ms and amplitude data ranging from 0.62 to 0.91 mV [20]. The Canadian team of Chen et al. worked with healthy participants with general characteristics similar to those of this project; they reported a mean amplitude of 0.66 mV and a latency of 6.54 ms, similar to our study [9]. Finally, in 2019, Vincent et al. evaluated 155 clinically healthy French individuals whose somatometry was almost identical to that of the population in our study and reported an average latency of 6.59 ms and an amplitude of 0.38 mV [13]. Although the amplitude difference between the LPN and RPN is significant, the CI range includes zero, so the possibility that there is no difference between the groups cannot be ruled out, and the effect size (Cohen’s d) is only moderate (*d* = 0.51). Table 4 shows the results of PNC measurements in several studies in different parts of the world. These discrepancies imply the existence of as-yet-unidentified population factors that may influence conventional PNC measurements. This underscores the necessity for conducting local studies to facilitate subsequent analysis of interpopulation differences, which can provide more precise guidance on the most suitable measurements to employ in clinical practice.

When comparing PNC measurements between sexes, we found a significant difference only in left phrenic nerve latency, with medians of 5.83 and 6.37 ms for women and men, respectively (*p* = 0.0348); however, the effect size (Cohen’s d) is small to moderate (*d* = 0.45). In the study by Imai et al., no significant differences were found in any of the phrenic nerve conduction parameters between men and women [22]. Although we observed greater latency in men, we did not find statistically significant differences in RPN latency and the effect size was small (d = 0.25). However, the CIs are very wide and indicate that the difference could be confirmed or denied, so both latency results (LPN and RPN) should be supported by results from a larger sample size. Although men presented a wider age range, their median age was significantly lower than that of women, and the findings of the linear regression analysis indicate a positive correlation between age and latency, thereby suggesting that the observed variations are not attributable to age differences. Several studies have reported a direct association between age and phrenic nerve conduction latency, even suggesting that it increases by 0.32 ms for each decade of life from the age of 60 [9,13,19,22], reaching average latency values of up to 8.25 ms from the age of 90 [22]; examples of studies that show a positive correlation between latency and age are those of Chen et al. (*r* = 0.73, *p* = 0.0001) [9], Mieret al. (*r* = 0.26 and *p* < 0.01) [19] and Vincent et al. (*r* = 0.38 and *p* < 0.001) [13]. Although we did not detect a positive and statistically significant correlation between age and PNC latency, which was also reported by Carvalho et al. and Davis et al. [12,20], the coefficients of correlation for both sides were greater (LPN *r* = 0.9415, CI [0.8987, 1.2334], *p* = 0.1695 and RPN *r* = 0.9208, CI [0.8639, 1.3671], *p* = 0.5206), suggesting that there is a high probability that latency may be affected by age but not yet statistically significant, this could be due to the small sample size of our study, which is reflected by the high dispersion of the observed values and the CI. Studies involving larger sample sizes are needed to prove positive correlation between age and PNC latency.

Thoracic circumference measurement has been associated with differences in PNC amplitude, as reported by Chen et al. [9], who reported *r* = 0.58, *p* = 0.005. Although the coefficients of correlation were higher in our study (r = 0.9297, CI [0.8787, 1.4153] and *r* = 0.9635, CI [0.9362, 1.1968]; for LPN and RPN, respectively), we did not find a statistically significant relationship between PNC amplitude and thoracic circumference. However, even with the strong coefficient of correlation for both phrenic nerves, suggesting a possible relationship between these variables, the non-significant difference and the amplitude of the CIs could be due to the small sample size of our study and the high dispersion of values in our patient sample, which could be confirmed in a study with a larger sample size.

Our study has several limitations that should be considered when interpreting the results. First, despite the importance of this study as it is the first to report the phrenic nerve conduction values of a sample of Mexican patients, these results cannot be generalized to the general Mexican population and only describe the population that regularly visits or lives around our hospital to a limited degree. In addition, the primarily descriptive nature of our study is not the most appropriate for drawing analytical conclusions; therefore, a study focused on finding these possible relationships, carried out through the formulation of formal statistical hypotheses and the subsequent calculation of the sample size, as well as an intentional search for possible differences, should be carried out, and we believe that the values found in our study could serve as a reference for a sample size calculation focused on answering these questions. Another important limitation is that, although the medical office′s ventilation is controlled by air conditioning at a fixed temperature of 24 °C, the ambient temperature was not precisely measured. The main importance of our research is that, to our knowledge, there are no studies with Mexican patients who show normal measurements of phrenic nerve amplitude and latency. Although our results cannot be generalized to the national level, they can serve as a basis for conducting a larger-scale multicenter study, taking into account factors such as possible differences associated with age groups and the integration of lung function studies, among others, and that can be used to measure reference values for our population of patients [24]. Conducting studies in multiple geographic areas of Mexico and subsequently analyzing and comparing them can provide greater certainty for generalizing the results.

## 5. Conclusions

This study provides a first suggestion of reference values for phrenic nerve conduction latency and amplitude in healthy Mexican adults. These normative data may assist clinicians in the evaluation of patients with suspected diaphragmatic paralysis, neuromuscular disorders, or unexplained respiratory failure. We observed significantly greater amplitudes in the right phrenic nerve and longer latencies in men for the left phrenic nerve; however, it is imperative to interpret these discrepancies, along with the rest of the findings, within the context of the study′s limitations and the modest sample size. No significant associations were found between latency and age or between amplitude and thoracic circumference; however, the correlation coefficient values indicate positive relationships between age and conduction latency, as well as between amplitude and thoracic circumference. This discrepancy could perhaps be attributed to the small sample size. As this is the first report of its kind in the Mexican population, larger multicenter studies are warranted to validate and expand these findings for broader clinical application.

## Figures and Tables

**Table 1:** General characteristics of the study sample.

Sample Size, *n* = 50	Median	±SD
Age (years)	43.64	14.87
Weight (kg)	69	9.23
Height (m)	1.69	0.07
BMI (kg/m^2^)	23.97	2.20
Thoracic perimeter (cm)	94.99	4.82

SD = standard deviation, BMI = body mass index.

**Table 2:** Comparison of PNC measurements: LPN vs. RPN.

	LPN, *n* = 50		RPN, *n* = 50		CI 95% *	Cohen’s d	*p*
	Median	±SD	Median	±SD			
Latency (ms)	6.13	1.23	6.07	1.72	[−0.6, 0.4]	0.04	0.7376
Amplitude (mV)	0.55	0.22	0.65	0.17	[0, 0.2]	0.51	0.0036

* = CI for the Mann-Whitney U test, ms = milliseconds, mV = millivolts, LPN = left phrenic nerve, RPN = right phrenic nerve, CI = confidence interval, SD = standard deviation.

**Table 3:** Comparison of PNC measurements, age, and thoracic perimeter between sexes.

	Female, *n* = 22		Male, *n* = 28		CI 95% *	Cohen’s d	*p*
	Median	±SD	Median	±SD			
Latency LPN (ms)	5.83	1.10	6.37	1.30	[0.1, 1.2]	0.45	0.0348
Latency RPN (ms)	5.83	1.53	6.27	1.86	[−0.7, 1.2]	0.25	0.8346
Amplitude LPN (mV)	0.61	0.27	0.50	0.15	[−0.2, 0]	0.5	0.2416
Amplitude RPN (mV)	0.65	0.18	0.64	0.16	[−0.1, 0.1]	0.06	0.7218
Age (years)	48.86	14.78	37	12.38	[−21, −4]	0.87	0.0027
Thoracic perimeter (cm)	93.92	4.09	95.83	5.24	[−5.1, 0.9]	0.41	0.2039

* = CI for the Mann-Whitney U test, ms = milliseconds, mV = millivolts, LPN = left phrenic nerve, RPN = right phrenic nerve, CI = confidence interval, SD = standard deviation.

**Table 4:** Measurements of the latency and amplitude of the PNC have been performed in some clinical studies over the last 50 years.

Study	Country	*n*	Age Range (Years)	Mean PNC Latency, ms (SD±)	Mean PNC Amplitude, mV (SD±)
Davis et al. [12]	England	18	20–61	7.7 (0.8)	0.16–0.50
Carvalho et al. [20]	Portugal	19	25–70	7.7 (0.92)	0.62–0.91
Chen et al. [9]	Canada	25	22–80	6.54 (0.77)	0.66 (0.20)
Similowski et al. [21]	France	7	21–35	RPN: 6.57 (0.97) LPN: 6.24 (0.72)	0.45–0.87
Imai et al. [22]	Japan	25	35–55	6.81 (0.75)	0.40 (0.17)
Vincent et al. [13]	France	155	25–79	6.59 (0.82)	0.38 (0.15)
Torrieri et al. [23]	Portugal	18	42–65	7.98 (0.78)	0.41 (0.09)
Our study	Mexico	50	18–75	6.10 (1.23)	0.60 (0.20)

SD = Standard deviation, PNC = Phrenic nerve conduction, RPN = Right phrenic nerve, LPN = Left phrenic nerve.

## Data Availability

The data presented in this study are available on request from the corresponding author due to privacy reasons.

## Abbreviations

The following abbreviations are used in this manuscript:

PNC	Phrenic nerve conduction
SD	Standard deviation
NC	Neuroconduction
CNS	Central nervous system
BMI	Body mass index
CI	Confidence interval
GCP	Good clinical practices
LPN	Left phrenic nerve
RPN	Right phrenic nerve
